# Observations on the Cases of Yellow Fever Received into the Marine Hospital, Charleston, from July, 1834, to November, 1838

**Published:** 1848-01

**Authors:** 


*Observations on the Cases of Yellow Fever received into the Marine Hospital, Charleston, from July,* 1834, *to November,* 1838. In accordance with your request that I would furnish you with a report of the cases of yellow or strangers' fever, which came under my observation during the period of my official attendance at the Marine Hospital in this city; I have submitted a huried, and I fear imperfect sketch of the cases. Careful and particular notes were taken on every one, the publication of which would be too lengthy for your journal. I commenced my career of attendance in the Hospital in July, 1834, and resigned in October, 1838—during this period there were 1129 cases of different diseases admitted. A few weeks after my duties commenced, many cases of what is commonly termed bilious remittent fever, from various miasmatic localities, were admitted; the success of my practice on these cases was unsatisfactory, and I felt discouraged. On the 9th September, the first case of yellow fever was admitted, and the last on the 2d October—the whole number admitted were 8. Period of the disease as follows: the statement of which is very important in a report of Hospital practice, where patients are often received when the disease is out of the reach of any remedy.

1 case was admitted on the 1st day of disease.
4 do do do do do 2d do do
3 do do do do do 3d do do

*Result*—the one admitted on the first day recovered; o those on the second day, three died; those on the third day, two died—making the mortality five out of eight. The termination of these cases, in connexion with other cases of fever the same season, I must confess, did not tend to strengthen my experience of the efficacy of my practice, which I had strong faith in, as every practitioner pursued it in a great measure, and it was the prominent doctrine taught in the schools of medicine—the professors and the text books promulgated it as the orthodox practice. I faithfully followed the rules laid down. I scarcely need detail the practice, as it would not be difficult for any of your readers to guess rightly. It consisted in a free use of the sampson of the materia medica, the *mercurial* "sine qua non" in all diseases, but especially the one under consideration, cathartics to the extent, that the chamber utensil must be frequently *filled* during the day and night, the contents of strong odor and highly bilious character to be kept for inspection at every visit, and for the gratification of the olfactory nerves, which were never offended, as the more odoriferous it was, the more effectually I was taught, was the medicine doing its duty.

Blood-letting, local and general, in some cases but very cautiously practised, as I had been repeatedly warned against that *awful bugbear*, *debility*, which "has made cowards of us all." Blood-letting never was repeated, and a small quantity was abstracted; nitrous powders, to act on the skin, was an essential item in the treatmet. After death, I sought for the cause, and I found that intense gastro-duodenits invariably existed in every instance, and if the disease lasted seven or eight days, nearly the whole tract of the intestines was inflamed, but more especially the ilium; the glands of Peyer and Brunner evidently so, and enlarged very visibly under the microscope. The liver presented no unusual appearance, notwithstanding Louis, and others, have asserted that it was yellow. The membranes of the brain were generally congested. In 1835, '36, and '37, no cases of yellow fever were admitted, but many cases of bilious remittent, which I treated with rather a less quantity of calomel, and a little more blood-letting, still fearing that some patient might pop off as soon as *I tied up the arm.* I was rather more successful, but not at all satisfied, and I felt that the treatment "was not as successful as I desired."

In 1838, the first case of yellow fever was admitted on the 23d June, and I determined to pursue the non-perturbating plan of practice. I was fully aware that I would receive encouragement from no one in the city of calomel and bile; but I reasoned that no practice could be less successful than the one I had pursued. I also knew that the non-mercurial practice had never been tried, as it was considered medical sacrilege to let any man, woman or child die of fever or any disease without the use of the wonderful "sub mur." I determined to pursue a plan of practice based on pathological principles. The first step I took was to expel from my mind the phantom debility, as I believed what was so called was caused by excessive action, not want of action; similar to the inability of locomotion, caused by intoxication; I did not believe that the liver was the "fons et origo" of the disease ; too much or too little bile did not alarm me, as I was aware that all the secreting organs were in the same condition. For these few reasons, (and many others which I will state more fully hereafter,) when the patients were admitted early in the disease, I bled freely, always in a recumbent position, as a person with a severe attack of fever cannot remain long in a standing or sitting posture, without feeling faint, and if the vein is opened in this position, this feeling is attributed to the loss of blood, even if but one oz. is taken, and it is apt to be said that the patient is bled ad deliquium. I allowed the blood to flow, until by the pulse I felt that the action of the heart was reduced. In looking over the notes of the cases, I find that the average quantity of blood abstracted from the patients who were admitted early in the disease to be about 50 oz.;* in one case when the determination to the head was very great indeed, I took 72 oz. at one bleeding.† Yellow fever is said to be a disease of one paroxysm; but I have observed evident remissions after free depletion by the lancet. Local depletion from the epigas. regions and back of the neck, was invariably resorted to—the principal medicine administered was castor oil, and, if the stomach was irritable, I found olive oil was more apt to be retained. Enemata of oil and molasses were generally used; when the skin was dry and hot the cold dash was used, and when disposed to be cool, the warm bath, with a large portion of salt dissolved in it. Iced mucilage constituted the drink of the patients. I have given you but a very crude and condensed sketch of the practice I pursued. In a future communication I hope to give you my views on the pathology of the disease, and the different remedies which have been used more fully. The following is an extract from my note book: first case of yellow fever was admitted on 23d June, 1838—total number of cases treated, 75.

* "Young Physic" and the European Dr. Samuel Dickson to the contrary notwithstanding.

† This patient was a strong robust man, I saw him soon after he was taken, he said, "Dr. if you don't make haste and bleed me, my head will burst or my heart will jump out of me." I have never felt arterial action so strong.

| Day of disease. | Cases admitted. | Cured. | Died. |
|---|---|---|---|
| 1st day, | 13 | 11 | 2 |
| 2d " | 28 | 24 | 4 |
| 3d " | 20 | 14 | 6 |
| 4th " | 5 | 1 | 4 |
| 5th " | 4 | 0 | 4 |
| 6th " | 3 | 0 | 3 |
| | — | — | — |
| | 73 | 50 | 23 |

Two cases were admitted in a dying state, one died four hours, the other eight hours after admission; no cases recovered after black vomit; one recovered after having vomited blood freely.

Since this period I have attended with success many cases of bilious and remittent fever by the same practice, and I am perfectly satisfied with the result.—*Southern Journal of Medicine and Pharmacy.*

